# The effectiveness and cost-effectiveness of community-based lay distribution of HIV self-tests in increasing uptake of HIV testing among adults in rural Malawi and rural and peri-urban Zambia: protocol for STAR (self-testing for Africa) cluster randomized evaluations

**DOI:** 10.1186/s12889-018-6120-3

**Published:** 2018-11-06

**Authors:** Melissa Neuman, Pitchaya Indravudh, Richard Chilongosi, Marc d’Elbée, Nicola Desmond, Katherine Fielding, Bernadette Hensen, Cheryl Johnson, Phillip Mkandawire, Alwyn Mwinga, Mutinta Nalubamba, Gertrude Ncube, Lot Nyirenda, Rose Nyrienda, Eveline Otte im Kampe, Miriam Taegtmeyer, Fern Terris-Prestholt, Helen A. Weiss, Karin Hatzold, Helen Ayles, Elizabeth L. Corbett

**Affiliations:** 1 Faculty of Epidemiology and Population Health, London School of Hygiene and Tropical Medicine, Keppel Street, London, WC1E 7HT UK; 2 Malawi-Liverpool-Wellcome Trust Clinical Research Unit, Blantyre, Malawi; 3 Population Services International, Lilongwe, Malawi; 4 Liverpool School of Tropical Medicine, Liverpool, UK; 5 World Health Organization, Geneva, Switzerland; 6 Zambart, Lusaka, Zambia; 7 Society for Family Health, Lusaka, Zambia; 8 Zimbabwe Ministry of Health, Harare, Zimbabwe; 9 Malawi Ministry of Health, Lilongwe, Malawi; 10 Population Services International, Harare, Zimbabwe; 11 Faculty of Public Health and Policy, London School of Hygiene and Tropical Medicine, Keppel Street, London, WC1E 7HT UK; 12 Faculty of Infectious and Tropical Disease, London School of Hygiene and Tropical Medicine, Keppel Street, London, WC1E 7HT UK

**Keywords:** HIV/AIDS, Self-testing, Malawi, Zambia

## Abstract

**Background:**

Knowledge of HIV status remains below target in sub-Saharan Africa, especially among men and adolescents. HIV self-testing (HIVST) is a novel approach that enables unique distribution strategies, with potential to be highly decentralised and to provide complementary coverage to facility-based testing approaches. However, substantial gaps in evidence remain on the effectiveness and cost-effectiveness of HIVST, particularly in rural settings, and on approaches to facilitate linkage to confirmatory HIV testing, prevention, and treatment services. This protocol describes two cluster-randomized trials (CRT) included within the UNITAID/PSI HIV Self-Testing Africa (STAR) project.

**Methods:**

Two independent CRTs were designed around existing reproductive health programmes in rural Malawi and rural/peri-urban Zambia. Common features include use of constrained randomisation to allocate health clinic catchment areas to either standard HIV testing (SOC) or SOC plus community-based distribution of OraQuick HIV Self Tests (Bethlehem, PA USA, assembled in Thailand) by trained lay distributors selected by the community. Community-based distribution agents will be trained (3-day curriculum) to provide brief demonstration of kit use and interpretation, information and encouragement to access follow up services, and management of social harm.

The primary outcome of both CRTs is the proportion of the population aged 16 years and older who tested for HIV within the 12-month intervention period. Secondary outcomes in both trials include lifetime HIV testing, antiretroviral therapy (ART) initiation and ART use. Circumcision status among males will be a secondary outcome in Zambia and clinic-level demand for ART will be a secondary outcome in Malawi. Outcomes will be measured using cross-sectional household surveys, and routine data extraction from participating clinics. Costing studies will be used to evaluate the cost-effectiveness of the intervention arm. Qualitative research will be used to guide distribution and explore reasons for testing and linkage to onward care.

**Discussion:**

The STAR-Malawi and STAR-Zambia trials will provide rigorous evidence of whether community-based lay HIVST distribution is an effective and cost-effective approach to increasing coverage of HIV testing and demand for follow-on HIV services in rural and peri-urban communities in sub-Saharan Africa.

**Trial registration:**

Clinicaltrials.gov, Malawi: NCT02718274, 18 March 2016; Zambia: NCT02793804, 3 June 2016. Protocol date: 21 February 2018.

## Background

Knowledge of HIV status remains low in many parts of sub-Saharan Africa, particularly among men and adolescents (aged 10–19) [1]. As a gateway to treatment and prevention services, HIV testing is key to achieving United Nation’s (UN) 2020 fast track targets [2, 3]. However, recent population-based data from Malawi and Zambia show that the percent of people with HIV (PHIV) who know their status is far lower than the 90% benchmark set by the UN, with only 73% of PLHV in Malawi and 67% of PLHIV in Zambia aware of their status in 2015 [4, 5]. Despite integration and decentralisation of facility-based HIV testing services in antenatal care, tuberculosis clinics and primary care, as well as community-based outreach to reach remote and under-served communities, knowledge of status remains low [6, 7]. To overcome these gaps and achieve the UN testing targets, novel and affordable approaches are needed.

HIV self-testing (HIVST) has potential to increase accessibility to and uptake of HIV testing, particularly among populations not well-served by existing HTS. In HIVST, individuals collect their own specimen, conduct their own test, and interpret their own result. In 2016, the World Health Organization (WHO) recommended HIVST as a way to expand HTS services, particularly to high risk and underserved populations [8]. Early studies in Malawi and elsewhere have shown HIVST to be acceptable, and that oral fluid HIVST kits can be performed accurately, especially when provided with a brief in-person or video demonstration along with instructions-for-use provided by the manufacturer [9–11].

HIVST may provide more readily affordable and sustainable community-based HTS than existing models [12, 13]. Cost-effectiveness was shown for a community-based HIVST study in urban Malawi [14, 15]. Despite the potential impact of HIVST, evidence concerning cost-effectiveness for rural communities is limited. Few data to inform evidence-based choices between different HTS options for encouraging timely linkage to confirmatory testing and prevention and treatment services.

The Unitaid/PSI STAR (HIV Self-Testing Africa) project was developed to strengthen the evidence base around the effective use of HIVST in sub-Saharan Africa. STAR includes the design, implementation, and evaluation of a variety of HIVST distribution models and post-test linkage strategies. STAR also promotes the development of regulatory and policy environments enabling distribution of quality-assured HIVST kits. This protocol describes two CRTs within the STAR project assessing the effectiveness and cost-effectiveness of community-based HIVST distribution models in Malawi and Zambia.

### Study objectives

The overall objective of these trials is to evaluate the effectiveness of distribution of oral-fluid HIVST kits by community-based distribution agents (CBDA) on uptake of HIV testing, including coverage of recent (within 12 months) and lifetime testing among the population ages 16 years and older, and on ART initiation. The Zambia study also assesses the impact of self-testing on men’s circumcision status, and the Malawi study evaluates differences between arms in linkage to ART (offered routinely under a “test-and-start” strategy by the Government of Malawi) through extraction of clinic-level ART initiation data. Formative research, consisting of qualitative studies and discrete choice experiments (DCEs), will inform the final intervention design, training materials, social harms definitions. Procedures for reporting social harms related to the intervention are informed by qualitative research in each setting. Qualitative research and DCEs will also be used to maximise preferences for service configurations offered for testing and linkage, and to understand participant experiences. Costing studies and a cost-effectiveness analysis of the interventions will also be conducted.

Specific hypotheses tested by the trials include that community-based distribution of HIVST kits will increase:

1. the proportion of the population who have tested for HIV within the past 12 months;
2. the proportion of the population who have ever tested for HIV;
3. the rate of ART initiations in clinics serving populations that received HIVST distribution.

## Methods and design

### Settings

CRTs will be conducted in rural settings in Malawi (Blantyre, Machinga, Mwanza and Neno districts) and rural, urban and peri-urban settings in Zambia (Ndola, Kapiri Mposhi, Lusaka and Choma districts). (Tables 1 and 2 provide additional information on the study sites).

**Table 1:** HIV STAR-Malawi study clinics

District	Clinic name	Clinic Catchment Population (2014–2015)	Total HIV tests (2014–2015)	Percent HIV-positive tests (Number of positive tests / total tested at clinic 100%)	Allocation
Blantyre (N)	Chikowa Health Centre	34,317	4128	8.0%	HIVST
Blantyre (N)	Dziwe Health Centre	18,430	1794	7.6%	SOC
Blantyre (N)	Lirangwe Health Centre	27,880	3268	14.4%	SOC
Blantyre (N)	Makata Health Centre Lunzu	37,000	3502	6.9%	SOC
Blantyre (S)	Madziabango Health Centre	15,000	2281	7.2%	HIVST
Blantyre (S)	Mpemba Health Centre	23,600	5027	10.9%	HIVST
Blantyre (S)	Pensulo Health Centre	19,172	706	9.8%	SOC
Blantyre (S)	Soche Maternity	16,208	784	13.6%	HIVST
Machinga	Chikweo Health Centre	82,581	6938	6.8%	HIVST
Machinga	Mangamba Health Centre	22,364	5344	6.1%	HIVST
Machinga	Mbonechera Health Centre	112,934	1195	7.6%	SOC
Machinga	Mkwepere Health Centre	19,317	2715	6.6%	SOC
Machinga	Namanja Health Centre	34,678	3930	6.8%	HIVST
Machinga	Nayuchi Health Centre	21,582	2642	11.5%	SOC
Machinga	Ngokwe Health Centre	40,778	7165	5.3%	SOC
Mwanza	Kunenekude Health Centre	12,085	2758	4.9%	HIVST
Mwanza	Thambani Health Centre	19,056	2561	5.0%	SOC
Mwanza	Tulonkhondo Health Centre	16,700	2261	4.1%	SOC
Neno	Chifunga Health Centre	11,250	1994	6.0%	HIVST
Neno	Ligowe Health Centre	15,025	2118	5.0%	SOC
Neno	Luwani Health Centre	4452	1027	5.3%	HIVST
Neno	Magareta Health Centre	7547	1550	3.4%	HIVST

**Table 2:** HIV STAR-Zambia study clinics

Pair	District	Community	Type of community (rural/peri-urban)	Distribution models	Population (2016)	Distance to district medical office in km	Allocation
1	Choma	Mbabala	Rural	Community, facility, VMMC	11,415	33	HIVST
1	Choma	Mapanza	Rural	Community, facility, VMMC	14,528	75	SOC
2	Choma	Batoka	Rural	Community, facility, VMMC	15,566	33	HIVST
2	Choma	Sikalongo	Rural	Community and facility	13,491	66	SOC
3	Kapiri Mphoshi	Chankomo	Rural	Community and facility	15,228	55	SOC
3	Kapiri Mphoshi	Nkole	Rural	Community, facility, VMMC	15,365	34	HIVST
4	Kapiri Mphoshi	St Pauls	Rural	Community and facility	7673	90	SOC
4	Kapiri Mphoshi	Mpunde	Rural	Community, facility, VMMC	15,694	48	HIVST
5	Lusaka	Ng’ombe	Peri-urban	Community, facility, VMMC	45,999	8	HIVST
5	Lusaka	Makeni	Peri-urban	Community and facility	51,264	9	SOC
6	Ndola	Lubuto	Peri-urban	Community, facility, VMMC	53,249	8	SOC
6	Ndola	Twapia	Peri-urban	Community, facility, VMMC	24,078	9	HIVST

### Study design

Using a parallel arm design, clinics will be randomly allocated either to receive community-based HIVST distribution in a defined catchment area, or to continue with usual clinic-based HTS (standard of care [SOC]). Clinic catchment areas will be randomized rather than individual testers because the intervention was designed to be delivered by community-based distributors. Individual-level randomization would therefore be inappropriate.

In Malawi, 22 rural primary health clinics will be randomized 1:1 to receive the HIVST intervention or SOC. Within the HIVST intervention arm, CBDAs will also be randomized 1:1 to provide either referrals to home-based ART initiation or the usual referrals to the clinic. Each clinic will contain a pre-defined, concentrated area of implementation, with two villages in each area selected for the baseline and endline household surveys, respectively.

In Zambia, 12 primary health clinics in 4 districts will be pair-matched within districts by distance to the district medical office and catchment area population, and randomized 1:1 to provide the HIVST intervention or SOC.

### Intervention and standard of care

Both trials will assess the effectiveness of community-distributed HIVST on HIV testing uptake using OraQuick HIV Self Tests (Bethlehem, PA USA, assembled in Thailand) including instructions-for-use translated by the manufacturer into local languages (Chichewa in Malawi; and Nyanja, Tonga, and Bemba in Zambia). The specificity and sensitivity of the test kit in a general rural population in sub-Saharan Africa will be assessed in separate studies [16]. HIVST kits will be stored and distributed by Population Services International (PSI)-Malawi or Society for Family Health (SFH)-Zambia. In both trials, CBDAs will be trained by PSI or SFH in how to explain the written self-test instructions, demonstrate correct use, and show examples of reactive and non-reactive tests to assist in reading results. Distributors will also be trained on appropriate test kit storage and information concerning linkage to confirmatory testing and ART for clients with reactive HIVST results or prevention for clients with non-reactive results. Clients will be offered the choice of testing with CBDA assistance or in private. CBDAs will also be trained in how to pre-empt, respond to and report social harm, including suicide, gender-based violence, or coerced testing. Individuals are eligible for HIVST if they are ages 16 years or older.

In Malawi, CBDAs will provide door-to-door community-based distribution of HIVST kits as well as access from their own homes, building on a pre-existing initiative providing reproductive health products. CBDAs will provide all clients who wish to test with: (1) information on the test and a demonstration on how to open and use the HIVST kit; (2) an envelope and short anonymous questionnaire to be completed by the client and returned with the kit; and (3) a self-referral form to facilitate linkage into HIV care and prevention services. CBDAs will distribute one test per resident who is interested in testing. CBDAs will visit clients after distributing the kit to enquire whether the kit has been used, collect the sealed questionnaire and used kit, and provide advice on referral to additional care if the client discloses a reactive HIVST result. Clients will also be able to drop used kits in locked boxes within each community, or return them to CBDAs at their homes.

CBDAs will be paid MWK 100 (USD 0.15) for each test kit distributed, and an additional bonus (MWK 50, or USD 0.08) for facilitating onward linkage to health services for clients with reactive HIVST results.

#### Malawi – Home-based ART intervention

In a second-stage randomisation, CBDAs working within clinic catchment areas allocated to the intervention (HIVST) will be randomly allocated to either no additional intervention, or HIVST plus the offer of home-based confirmatory testing, WHO staging, TB symptom screening and initiation of HIV care including a 4 week supply of ART and cotrimoxazole and a government ART patient card for service continuation at the nearest clinic. HIVST clients will need to confide their reactive self-test result with the CBDA, who will then arrange a single home visit by PSI nurses within seven days. This builds on a successful urban intervention of similar design [17].

#### Zambia – HIVST intervention

In Zambia, multiple modes of HIVST distribution will be provided to the catchment population of each intervention facility in addition to a similar CBDA model to Malawi. Healthcare workers will distribute HIVST within health facilities, while CBDAs will visit households to dispense HIVST kits. Voluntary male medical circumcision (VMMC) mobilizers employed by PSI are associated with some but not all facilities, and in intervention areas will distribute self-test kits as part of their activities. Distribution modes for each clinic are listed in Table 3. As in Malawi, clients who agree to HIVST will receive a demonstration of kit use; an envelope for the used kit and anonymous self-complete questionnaire to return with kit; and a self-referral card to the health facility to assist with linkage to confirmatory testing and care as needed. Boxes to return used self-test kits will be located at each facility as well as in public areas in the community, and used kits can be returned to CBDAs.

**Table 3:** Primary and secondary outcomes of HIV STAR-Zambia and HIV STAR-Malawi impact evaluations

Outcome	Trial	Data source
Primary outcome – community HIVST distribution		
Tested for HIV within past 12 months	Malawi, Zambia	Self-report, endline household survey
Secondary outcomes – community HIVST distribution		
Ever tested for HIV	Malawi, Zambia	Self-report, endline household survey
ART initiation 6 months and 12 months after distribution begins	Malawi, Zambia	Routine data extracted from clinic
Current ART use	Zambia	Self-report, endline household survey
Circumcision within past 12 months (men only)	Zambia	Self-report, endline household survey
Primary and secondary outcome – home-based distribution (Malawi only)		
Primary: Disclosure of a positive results to the CBDA during months 1 to 12 of intervention	Malawi	CBDA post-test log book
Secondary: ART initiation between months 1 and 12 of the intervention	Malawi	Routine data extracted from clinic

CBDAs will be recruited from the communities in which they will work, and will have previously worked with the health facilities included in the impact evaluation. CBDAs and VMMC mobilisers will be paid using a performance-based structure (ZMW 5/distributed kit and ZMW 2.5/used kit returned, or USD 0.56 and 0.28).

#### Standard of care (SOC)

In facilities allocated to the SOC arms, health facilities will provide HIV testing and ART initiation as dictated by national guidelines. In Malawi, pre-existing reproductive health CBDAs will continue providing services in standard of care areas.

### Trial participants and procedures

**Fig. 1:**
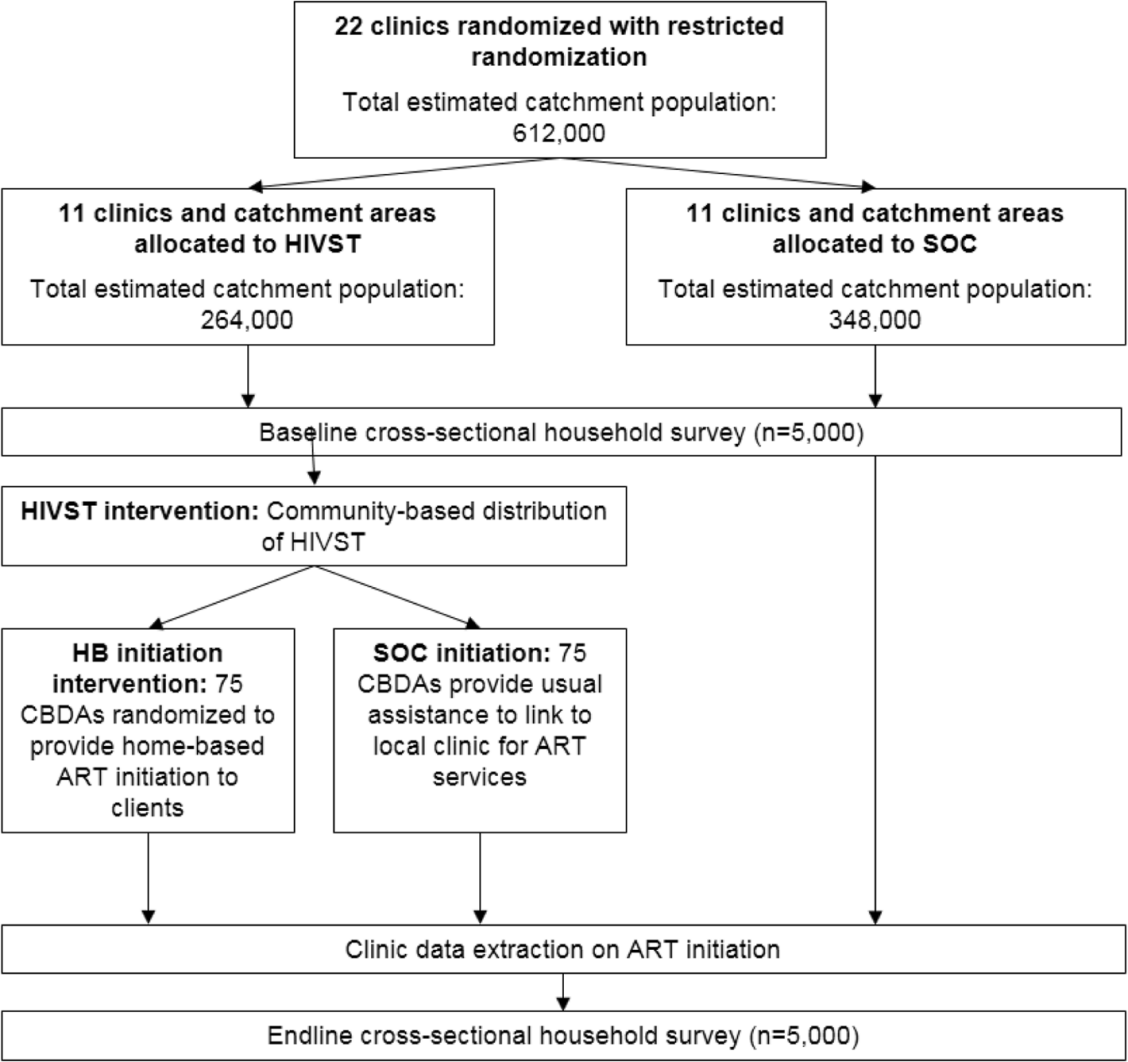
Flow chart, HIV STAR-Malawi

**Fig. 2:**
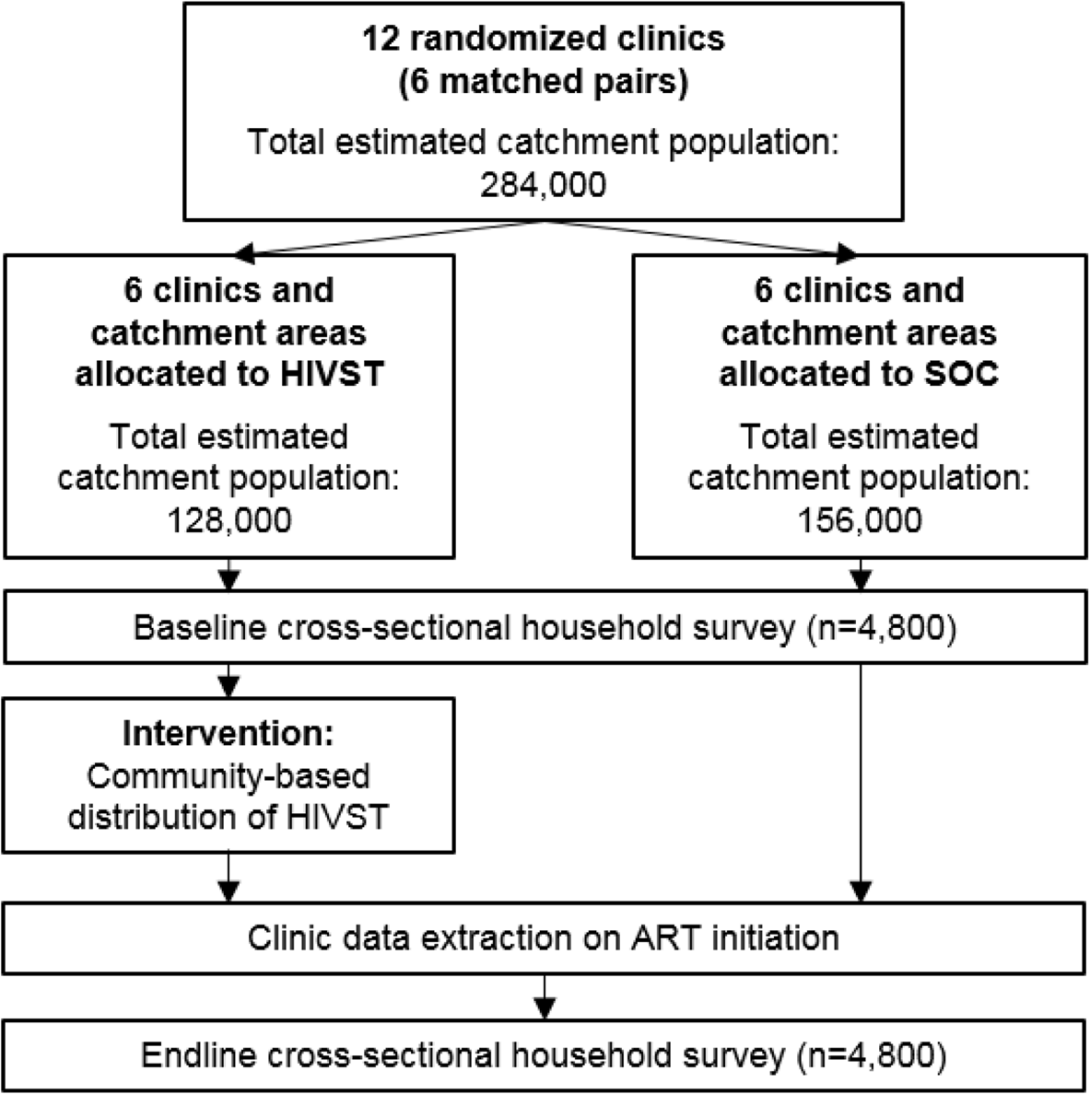
Flow chart, HIV STAR-Zambia

The trial flow charts (Figs. 1 and 2) illustrate the recruitment processes used during the two CRTs. In Malawi, all clinics offering ART in the study districts were eligible for inclusion. In Zambia, there were 109 clinics in four districts where HIVST would be distributed. Clinics hosting other HIV-related programs or that did not offer ART were not eligible for inclusion. Clinics were matched based on size of population served and, for peri-urban sites, distance from urban centre.

### Data collection

Repeated cross-sectional surveys will be used to evaluate the effectiveness of the intervention. Household survey data will be collected at baseline, before the intervention starts, and after at least 12 months of intervention delivery. Household surveys also include DCEs and questions on costs of testing for use in economic analyses. Contents of the baseline and endline surveys are online at: http://hivstar.lshtm.ac.uk/protocols/). In addition, process data on the number of tests distributed and used will be collected from individuals accepting HIVST by CBDAs and in questionnaires completed by clients themselves. Qualitative data will be collected for formative research and process evaluations. Table 4 provides an overview of data collected for the STAR-Malawi and STAR-Zambia impact evaluations.

**Table 4:** Overview of data collected for HIV STAR-Malawi and HIV STAR-Zambia impact evaluations

Data type	Data collected		
	Pre-intervention	During intervention period	12 months after intervention begins
Household survey	• Ever tested • Tested within past 12 months • Current ART use • Self-reported circumcision status		• Ever tested • Tested within past 12 months • Current ART use • Self-reported circumcision status
Clinic data extraction	ART initiations from each study clinic by sex, age, self-testing status		
CBDA M&E data		• Number of kits distributed • Sex and age of persons receiving kits • Barcode on test kit to link with returned kit	
Client-returned kit		• Used test kit • Self-completed questionnaire with test result, client sex, age • Barcode linking to CBDA M&E data	
Qualitative data	• Situational analysis and community mapping (ZM, MW) • Cognitive interviewing on IFUs (ZM, MW) • Formative research on attitudes and preferences toward self-testing (ZM)	• Process evaluation (ZM)	
Economics data	• DCEs to understand preferences for testing and linkage • Costs of health facility based HIV testing at baseline		• Costs of HIV testing, including self-test distribution models

#### Malawi – Survey data collection

**Fig. 3:**
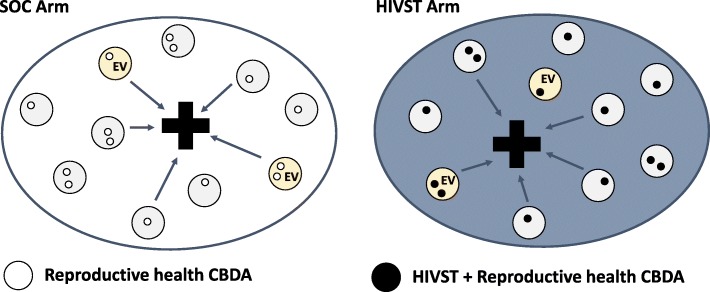
Diagram of Malawi clinic area

Evaluation data will be collected from selected villages within the implementation area of each clinic (Fig. 3**)**. Eligibility requirements for the evaluation villages will include:

- Location within the catchment area of an eligible ART clinic, with the clinic acting as the most dominant source of ART for the village.
- Presence of at least one active reproductive health CBDA prior to the study period.
- Population of at least 250 adults per village.
- Road access for most/all of the year.
- Sufficient distance and separation from administrative boundaries and other intended evaluation villages to minimise contamination between HIVST and control villages and missed linkage events from seeking HIV care at a clinic not included in the evaluation.
- Village delineation by natural boundaries (e.g., rivers, roads, forests, etc.)

Within evaluation villages, all households will be enumerated and a variable fraction will be surveyed to ensure that approximately 300 individuals within households will be included. Within selected households, all eligible household members ages 16 years and older will be surveyed with a short questionnaire including sociodemographic, testing, and sexual behaviour, and 20% of surveyed individuals will receive an extended questionnaire with added questions on HIV care and questions for economic evaluation. Baseline and endline surveys will be administered in different communities within each clinic catchment area.

#### Zambia – Survey data collection

Evaluation data will be collected from an area outlined using a radius of approximately 3.8 km around a central point within the intervention and control areas. Within this area, blocks of 20–30 households will be defined using mapping software and numbered in a spiral sequence. Blocks to be visited at baseline will be determined using a random number generator. Within each randomly selected block, all households will be visited and all eligible household members ages 16 years and older asked to participate in the household survey. An independent sample of blocks will be generated for the baseline and endline surveys.

### Power calculations for survey data

#### Malawi

The survey sample size was calculated to ensure sufficient power to identify a difference in the primary outcome between the HIVST intervention and standard care arms for the first stage randomization. The calculation accounts for clustering by incorporating the cluster coefficient of variation (*k*) using methods outlined in Hayes and Moulton [18]. The average cluster size was estimated to be 250–500 individuals, which is the typical size of a rural village based on previous experience working in Malawi, and a sample of 250 participants per cluster was used for the sample size calculations. We assume that the cluster coefficient of variation (*k*) was 0.25. Using 2010 Demographic and Health Surveys (DHS) data, the baseline proportion of adults tested in the last 12 months was estimated at 25% to 40% and ever tested at 42% to 60%. Using these assumptions, 11 clusters per arm will allow us to have 80% power to detect a 30% relative difference in recent HIV testing and a 45% relative difference in lifetime HIV testing between arms of the first stage randomization with α = 0.05.

#### Zambia

As in Malawi, the survey sample size was calculated to ensure sufficient power to identify a difference in the primary outcome between those receiving the HIVST intervention and those receiving standard care, and uses similar methods. We estimated that the cluster coefficient of variation (k) was 0.2, and that cluster populations were larger than in Malawi. Using 2013–2014 DHS data, baseline rates for adults tested in the last 12 months is estimated to be between 28.6–57.1% (lower in men than women). For this sample calculation, we will assume a testing rate of 50% in the standard of care arm. For a two-sample comparison of matched proportions across 6 pairs of matched communities, we will have 80% power to detect a 50% relative difference in recent HIV testing if we recruit around 400 respondents per community, or 4800 respondents in total.

### Randomization

The Malawi trial includes two stages of randomization. In the first stage, clinic catchment areas will be allocated to HIVST or standard of care arms using restricted randomization. To ensure that intervention and standard of care clinics are evenly distributed geographically across the study area, we will restrict the number of intervention clinics in each district, with larger districts to include at least 35% intervention clinics. We will balance arms in terms of number of testers, proportion of positive tests, and total catchment population across clinics. The study statistician (MN) provided a list of 1000 eligible randomization combinations to the study team and the final randomization scheme was selected in a public ceremony in Blantyre, Malawi on 21 March 2016.

In the second-stage randomization, we will randomize all CBDAs providing HIVST to home-based initiation of ART or standard of care at a 1:1 ratio. CBDAs will be randomized in blocks of varying sizes from 2 to 12 CBDAs, with the distribution of block sizes following Pascal’s triangle (i.e., 1:5:10:10:5:1).

In Zambia, clinics will be randomized 1:1 within matched pairs to receive the HIVST intervention or standard of care. Randomization was conducted by the statistician (BH) and was completed on 24 June 2016.

### Outcome evaluation and measurement

#### HIVST intervention

The primary outcome of the HIVST evaluation in both Malawi and Zambia is the proportion of village residents (16 years and above) who tested for HIV within 12 months after the beginning of the intervention period. Outcome data (numerators and denominators) will be collected during an endline household survey.

Secondary outcomes for the Malawi study include the proportion who report ever HIV testing, and the proportion initiating ART at clinics for participant residents in evaluation areas during months 1 to 12 of intervention. Outcome data (numerators and denominators) for ever testing will be collected during an endline household survey. ART initiation data will be extracted from clinic records at 3-month intervals, and the allocation status of each individual in the clinic records will be ascertained using the client’s village of residence. Denominator data will be village adult population counts.

Secondary outcomes for the Zambia study include reported HIV testing during the intervention period, reported lifetime testing for HIV, self-reported current ART use, ART initiation, and self-reported recent circumcision for men. Data to measure these HIV testing, current ART use, and recent circumcision will be collected in the household endline survey. ART initiation will be measured using routine facility data on ART initiations.

#### Malawi – Home-based ART intervention

The primary outcome for the second-stage randomisation of CBDAs to home-based initiation or clinic referral will be the number of participants (16 years and older) disclosing a positive result to the CBDA during months 1 to 12 of the intervention. Status disclosure is captured through returned self-completed questionnaires by the CBDAs.

The secondary outcome for this second-stage randomisation will be ART initiation rates for participants (16 years and older) during months 1 to 12 of intervention. Data to measure ART initiation will be collected in two different ways: for the HIVST testing intervention, data will be extracted from routine facility ART records as for Zambia. For the home-initiation versus facility-initiation linkage intervention, ART initiations will be identified through collection of “self-referral forms” provided to all clients that include CBDA identification information and data on the type of HIV care received. In the SOC arm, self-referral forms will be collected from health facilities. In the home-based-initiation arm, self-referral forms are collected from health facilities or the study nurse providing HIV care at home. Counts of self-referral forms will be compared to calculate differences in ART uptake across arms.

### Blinding

Because of the nature of the interventions, neither clients, distributors nor data collectors, will be blinded to the allocation status. However, analyses will be conducted on data with identification arm allocation removed, and the trial statisticians (MN, KF, BH) will be un-blinded only after data have been finalized.

### Data managem**e**nt

Quantitative data will be captured using electronic devices (tablets). In Malawi, program data will be extracted onto optical character recognition forms and processed into a dedicated database. Incoming electronic data will be checked regularly for errors, with supplemental training provided to field staff if required. All quantitative data will be cleaned and analysed using Stata 14 or 15 [19]. All participants will be assigned a study ID number. Their names will not be linked except through paper-based recruitment logs, which will be stored in locked cupboards. Similarly, in Zambia, quantitative client-level program data will be captured using electronic devices by CBDAs onto a platform based on Open Data Kit (ODK), an open source platform that allows for data collection through the use of mobile devices in real time and offline. From the CBDA, data will be uploaded to a central computer at SFH Head Quarters via the internet on a weekly basis, and data will be password-protected to ensure confidentiality. No client names will be collected, with linkage of client data instead using barcoded unique identifiers.

Qualitative data will be recorded in two forms – observational notes and digital audio recordings – and cross-referenced for accuracy. The audio file will be transcribed verbatim, and then will then be translated into English. All data will then be transferred to and coded using a qualitative data analysis software package, such as NVivo 10 [20].

As described elsewhere [21], data on costs will be extracted from project, PSI, and Ministry of Health records of expenditure including transport and training, unit costs, and salaries. Time and motion studies will be used to estimate the proportion of time spent on HTS by staff with multiple duties. Patient direct and indirect costs incurred to access HIV testing will be identified by interview at baseline, and for HIVST by interview at endline, surveys. Anonymized data will be made available in a public repository after the project is completed and findings disseminated.

### Fidelity of intervention and process evaluation

Quantitative monitoring and evaluation will be used together with qualitative process evaluation data to assess the following questions:

- ***Have HIVST kits been distributed in communities, and to whom?*** This will be assessed quantitatively using monitoring and evaluation data collected by CBDAs, as well as information provided by clients on the self-completed questionnaire returned with the used test kit. This will be used to assess the volume of distribution in different areas and demographics of recipients, and whether these characteristics changed over time.
- ***Have people in the intervention communities received and used HIVST kits?*** This will be assessed quantitatively using the self-completed questionnaire returned with the used test kit and the endline household survey by asking whether households were visited by CBDAs and received HIVST kits. These data will examine whether the intervention reached the expected population. Households in both intervention and SOC arms will be asked about CBDA visits to assess contamination.
- ***Do potential test users know how to receive care or prevention services?*** This will be assessed within the household survey at baseline and endline, and will be important for understanding whether clients could link to additional care following testing.
- ***Would test users recommend self-testing to their family or friends?*** This will be assessed within the endline household survey, and will be used to understand whether self-testing was a generally positive experience.

A qualitative process evaluation will also be conducted for each impact evaluation, following up on the issues described above. Qualitative research related to the trial is discussed in greater detail below.

### Reporting of social harms and adverse events

While HIV testing programs are well-established in these settings, concerns remain about the possibility of HIVST leading to harmful reactions from self-testers, their partners, or their families. We will assess social harms (including all forms of gender-based violence, any physical injury or related hospitalization and death due to assault) related to the self-testing intervention using a community reporting system established in each study cluster. Adverse events (AE) and serious adverse events (SAE) will be monitored for both trials.

In Malawi, social harm and AEs disclosed by clients to CBDAs will be recorded and followed up by PSI staff and local researchers. Active community-based reporting systems, with members pre-identified through social mapping, will also be established in the evaluation villages to report social harms. In Zambia, social harm and AEs will be tracked within a reporting system including CBDAs, local health clinics, community leaders and CBDA supervisors. CBDAs will be asked to follow up with clients experiencing serious events related to HIVST as well as any action taken. AEs will be categorized by severity, with SAEs including death, hospitalization, or violent assault within 30 days of a reactive HIV self-test. SAEs will be reported immediately to the principal investigator, while other AEs will be logged and reported regularly to the study team and technical advisory group (TAG).

In addition, we will use questions within the household survey on recent testing experiences to assess whether any HIV tests were coerced, whether clients regretted testing at the time of the test or at the time of the survey. These questions will be asked for all recent HIV tests, including both HIVST and standard HTS. We will also ask women participating in the survey about experiences with IPV at baseline and endline. Finally, we will assess the prevalence of perceived HIV-related stigma in the community at baseline and endline, to assess whether these beliefs change as a result of the intervention.

### Trial analysis

All analyses will be completed in Stata 14, on an intention-to-treat basis, and will use methods appropriate for CRTs with a small number of clusters [18]. Reporting will conform to the 2010 CONSORT statement as applicable to cluster randomized trials [22].

Analyses will be conducted separately by country. Initial analyses will compare characteristics of households and respondents across arms at baseline. These baseline analyses will be completed before analysis of endline data begins. Characteristics of households and respondents at endline will be summarized by arm, so that imbalances can be accounted for in our final analysis. Missing data will be examined for each variable and for each cluster or individual participant. A systematic assessment of missingness will be conducted to ascertain the reason and possible mechanism for missing data by identifying the quantity of missing data and patterns within the data. Missingness will be examined by cluster and between randomised arms to assess for systematic biases.

Primary and secondary trial outcomes will be compared using cluster-level unadjusted and adjusted means of recent testing. The analysis will give each cluster equal weight. For the unadjusted analysis, the overall prevalence of the outcome for each cluster will be calculated, and a log transformation will be applied to the prevalence for each cluster if needed. If there are any clusters with no events, one event will be added to all clusters so that the log transformation can be conducted. The mean and standard deviation of the log prevalence will be used to obtain the geometric mean and associated 95% confidence intervals for each trial arm.

For the adjusted analysis, a two-stage approach will be used. First, a logistic regression model including individual-level adjustment factors but not trial arm will be fitted, and predicted probabilities from this model used to estimate the ratio of observed to expected outcome events for each cluster. This ratio will be log transformed, and a t-test of the difference between logs by arm will be used to estimate the prevalence ratio, 95% confidence interval, and *p*-value. For both unadjusted and adjusted analyses, we will report risk ratios and risk differences. If adjustment for cluster-level factors is considered necessary, this will be conducted at the second stage using linear regression of the log ratio of observed and expected values on arm and cluster level factors, with appropriate adjustment for the degrees of freedom. The adjusted analysis will serve as the primary analysis of each trial. Sensitivity analysis for the primary outcome of recent test for HIV will be carried out by comparing complete case analysis results with those where missing outcome status are re-classified as yes and no.

### Economic analyses

#### Costing and cost effectiveness studies

The costing analysis will estimate the societal-level costs of community distribution of HIVST, both from the perspective of the health provider and the user, and will compare the costs of community HIVST distribution with standard HTS. The costing study will feed into estimates of cost-effectiveness, which will be projected on different time scales and population levels.

Full financial and economic costs from the providers’ perspective will be collected from PSI/SFH and from public health care facilities. User costs of accessing existing and new forms of HIV testing and linkage to care will be gathered using the extended baseline and endline questionnaires. Costing tools will be used in conjunction with service-related financial and activity reports in order to determine the unit costs of providing HTS and subsequent HIV care. We will also carry out detailed micro-costing, including time and motion studies, to clinics assigned to the intervention and control arms of the impact evaluation. This will help to identify instances of reduced crowding in ART clinics due to HIVST decentralisation. Gathered costing data will be used to conduct an economic evaluation, using decision-analytic modelling, to compare the costs of the different HIVST models to standard HTC models. Key outcomes will include the incremental cost per Disability Adjusted Life-Year (DALY) averted, which will allow the cost-effectiveness of HIVST and linkage to care models to be determined.

#### Discrete choice experiments

This study will administer two DCEs within baseline household surveys in Malawi and Zambia to inform HIVST implementation strategies. The first DCE will investigate preferences regarding delivery of HIVST relative to standard-of-care HTS, while the second DCE will evaluate preferences for linkage to onward HIV testing and care services [8]. We will sample approximately 500 participants for each DCE to allow for analysis of relative strength of preferences among the general population as well as among key sub-groups such as men and adolescents [23]. Participants will be randomly allocated to one of the two DCEs. We estimate HIV prevalence of 8.8% and 13% respectively in Malawi and Zambia [24, 25]. Respondents reporting HIV positive statuses were administered the linkage DCE. To elicit meaningful responses, the interviewer will present the HIV self-test kit to respondents and demonstrate the self-testing process at the beginning of the DCE.

Our analysis will include a simple multinomial logit and more complex choice models, such as mixed multinomial logit, latent class model and generalized multinomial logit model, to explore preference heterogeneity [26]. Socio-demographic characteristics and HIV-related experience will be explored to understand how they may influence respondents’ preferences.

### Qualitative research

Qualitative research related to the CRTs will include both exploratory and explanatory research as well as triangulation of findings across quantitative and qualitative methods. In the initial formative phase exploratory research will include an analysis of preferred self-test distribution models by age and sex to create an understanding of the concerns and preferences of various typical testers or ‘archetypes’. Results from preliminary analysis will be communicated to and discussed with implementers designing communications and marketing strategies as well as distribution models to shape delivery of self-tests. The formative research will also identify definitions and interpretations of harm among anticipated users, enabling us to better understand language around harm and coercion and to report harms using locally understood definitions. In-depth interviews with participants from the target populations will assess users’ cognitive understanding of instructions for use and ability to conduct tests, enabling us to develop support materials that optimise HIVST performance. Findings from these ‘cognitive interviews’ will also underpin the development of a training curriculum for distributors that addresses community concerns, demonstrates correct use of kits, and supports onward linkage. Finally, key informant interviews will be used to identify processes for and barriers to regulation of HIVST by national governments. As the study progresses we will undertake additional interviews with individuals and communities who have used HIVST to describe their experiences and help to probe behaviours around uptake, linkage, and coercive testing.

To facilitate cross-country qualitative research, we will establish an inter-country network of qualitative researchers (the qualitative research network) in STAR. This will allow for a robust analytical approach to the inter-country analysis of the qualitative findings that is interactive, collaborative and rooted in a strong understanding of the local contexts. Common data collection tools, sampling and coding frames will be developed iteratively through multiple rounds of joint discussion and analysis will thus feed into and be informed by the larger trial as an essential part of trustworthiness. We will use a framework approach to our qualitative analysis that will allow for both deductive and inductive themes to be captured and inform the larger trial at various stages as outlined above.

### Trial management

The trials will be overseen by an independent TAG, composed of up to six members who will be experts in research, health systems, policy, human resources and community health. The TAG meets semi-annually to review summaries of survey and monitoring and evaluation data, review any reports of severe adverse events or other social arms, and to assess the progress of these impact evaluations and other STAR research projects more generally. Both impact evaluations are short, pragmatic evaluations, and HIV self-testing is an established intervention. We do not expect substantial harms to result from the intervention, and will not establish a separate data safety monitoring board..

### Interim analysis and stopping rules

Interim survey data will be collected in intervention locations only to assess uptake of the intervention. These data will be collected between 4 and 6 months after the beginning of the intervention. Summaries of process and survey data will also be reviewed by the TAG. Frequencies of key process and outcome indicators will be distributed internally and to the donor. Because the intervention period will be brief and because we expect that there will be no substantial adverse impacts of the intervention, we will not establish a stopping rule for either study..

## Discussion

The STAR-Malawi and STAR-Zambia trials will provide rigorous evidence of whether community-based HIVST distribution is effective at increasing HIV testing uptake in rural and peri-urban communities in sub-Saharan Africa. Results from both CRTs and other STAR research will be used by local ministries to develop plans for self-testing in national HIV testing plans, and will be integrated into updated guidance for implementation of HIVST programmes by the WHO and other international organizations.

## Abbreviations

AE: Adverse events
ART: Anti-retroviral therapy
CBDA: Community-based distribution agents
CONSORT: Consolidated standards of reporting trials
CRT: Cluster randomized trial
DCE: Discrete choice experiments
DHS: Demographic and health survey
HIV: Human Immunodeficiecy Virus
HIVST: HIV self-testing
HTS: HIV testing services
IPV: Intimate partner violence
MWK: Malawi Kwacha
ODK: Open data kit
PHIV: People living with HIV
PSI: Population Services International
SAE: Severe adverse event
SFH: Society for Family Health
SOC: Standard of care
STAR: Self-Testing AfRica
TAG: Technical advisory group
USD: United States Dollars
VMMC: Voluntary Medical Male Circumcision
WHO: World Health Organization
ZAMRA: Zambian Medical Regulatory Authority
ZMW: Zambian Kwacha
